# Clinical factors associated with severity in patients with inflammatory bowel disease in Brazil based on 2-year national registry data from GEDIIB

**DOI:** 10.1038/s41598-024-54332-1

**Published:** 2024-02-21

**Authors:** Renata de Sá Brito Fróes, Adriana Ribas Andrade, Mikaell Alexandre Gouvea Faria, Heitor Siffert Pereira de Souza, Rogério Serafim Parra, Cyrla Zaltman, Carlos Henrique Marques dos Santos, Mauro Bafutto, Abel Botelho Quaresma, Genoile Oliveira Santana, Rafael Luís Luporini, Sérgio Figueiredo de Lima Junior, Sender Jankiel Miszputen, Mardem Machado de Souza, Giedre Soares Prates Herrerias, Roberto Luiz Kaiser Junior, Catiane Rios do Nascimento, Omar Féres, Jaqueline Ribeiro de Barros, Ligia Yukie Sassaki, Rogerio Saad-Hossne

**Affiliations:** 1 Gastromed - Department of Gastroenterology and Endoscopy, Rio de Janeiro, Brazil; 2 Department of Life Science, Bahia State University (UNEB), Salvador, Brazil; 3 Department of Proctology, Kaiser Hospital Dia, São José do Rio Preto, São Paulo Brazil; 4 Department of Clinical Medicine, Hospital Universitário Clementino Fraga Filho, Universidade Federal do Rio de Janeiro, Rio de Janeiro, Rio de Janeiro Brazil; 5 Department of Surgery and Anatomy, Ribeirão Preto Medical School, University of São Paulo, Ribeirão Preto, São Paulo Brazil; 6 Department of Surgery, Hospital Universitário Maria Aparecida Pedrossian, Campo Grande, Mato Grosso do Sul Brazil; 7 Department of Gastroenterology, Instituto Goiano de Gastroenterologia, Goiânia, Goiás Brazil; 8 Universidade do Oeste de Santa Catarina - UNOESC - Department of Health Sciences, Joaçaba, Santa Catarina Brazil; 9 Department of Medicine, Federal University of São Carlos - UFSCar, São Carlos, São Paulo Brazil; 10 Department of Proctology, Hospital Universitário João de Barros Barreto-UFPA, Belém, Pará Brazil; 11 Department of Gastroenterology, São Paulo Federal University, São Paulo, São Paulo Brazil; 12 Department of Proctology, Hospital Universitário Júlio Müller, Cuiabá, Mato Grosso Brazil; 13 Department of Internal Medicine, Medical School, São Paulo State University (Unesp), Botucatu, São Paulo CEP 18618-970 Brazil; 14 Department of Surgery, Medical School, São Paulo State University (Unesp), Botucatu, São Paulo Brazil

**Keywords:** Inflammatory bowel disease, Registry, Epidemiology, Severity, Ulcerative colitis, Crohn’s disease, Crohn's disease, Ulcerative colitis

## Abstract

The Brazilian Organization for Crohn's Disease and Colitis (GEDIIB) established a national registry of inflammatory bowel disease (IBD). The aim of the study was to identify clinical factors associated with disease severity in IBD patients in Brazil. A population-based risk model aimed at stratifying the severity of IBD based on previous hospitalization, use of biologics, and need for surgery for ulcerative colitis (UC) and Crohn’s Disease (CD) and on previous complications for CD. A total of 1179 patients (34.4 ± 14.7y; females 59%) were included: 46.6% with UC, 44.2% with CD, and 0.9% with unclassified IBD (IBD-U). The time from the beginning of the symptoms to diagnosis was 3.85y. In CD, 41.2% of patients presented with ileocolic disease, 32% inflammatory behavior, and 15.5% perianal disease. In UC, 46.3% presented with extensive colitis. Regarding treatment, 68.1%, 67%, and 47.6% received biological therapy, salicylates and immunosuppressors, respectively. Severe disease was associated with the presence of extensive colitis, EIM, male, comorbidities, and familial history of colorectal cancer in patients with UC. The presence of Montreal B2 and B3 behaviors, colonic location, and EIM were associated with CD severity. In conclusion, disease severity was associated with younger age, greater disease extent, and the presence of rheumatic EIM.

## Introduction

The incidence and prevalence of inflammatory bowel disease (IBD), including Crohn´s disease (CD) and ulcerative colitis (UC), are changing over the decades^1–3^. Recent studies have described the global epidemiological trends in incidence and prevalence, which can be stratified into four epidemiological stages: emergence of new cases, acceleration in incidence, compounding prevalence, and prevalence equilibrium^4^.

Developing countries in Latin America, Asia, and Eastern Europe have shown an increase in IBD cases, which could be attributed to an occidental lifestyle due to increased exposure to ultra-processed food, tobacco, sedentarism, stress, and air pollution^2,4–6^. In 2020, Salgado et al.^7^ published an article that considered the most important risk factors of IBD in the Brazilian population. Predictive factors for CD and UC were female sex (odds ratio [OR] 1.31; OR 1.69), low monthly family income (OR 1.78; OR 1.57), fewer cohabitants (OR 1.70; OR 1.60), absence of vaccination (OR 3.11; OR 2.51), previous history of bowel infections (OR 1.78; OR 1.49), and family history of IBD (OR 5.26; OR 3.33). IBD societies worldwide are alert to the importance of preventive behaviors in changing IBD’s emergence and healthcare costs related to IBD^8^.

However, there is still a lack of high-quality national population-based epidemiological data from newly industrialized countries. To date, there is a lack of detailed national-level clinical information on IBD in Brazil, including disease factors associated with severity or poor outcomes of the disease, making it difficult to compare data with those of other countries and across diverse regions of Brazil. The early identification of factors associated with a worse prognosis allows the stratification of patients according to severity and the indication of an early effective treatment, avoiding disease complications and disability, thus contributing to better disease control and health-related quality of life^9,10^.

The IBD National Patient Registry is an initiative of the GEDIIB (Brazilian Organization for Crohn's Disease and Colitis) that aims to survey the epidemiological profile of patients with IBD by creating a centralized registry with data on patients monitored in public and private healthcare services. This study aimed to characterize the profile and identify the clinical factors associated with disease severity in patients with IBD in Brazil.

## Materials and methods

### Study design

This cohort study was conducted between August 2020 and August 2022. Data obtained from medical records and/or from patients during regular follow-up visits were registered on the REDCap data platform. Participants of IBD Public and Private Centers previously approved in their Ethical Committee, who fulfilled the inclusion criteria, were subsequently recruited (random sampling method). Sample size was calculated considering a Brazilian epidemiological study^3^ with an estimation of at least 1000 patients in this study. The inclusion criteria were confirmed diagnosis of IBD and agreement to participate in the study. IBD diagnosis was based on established clinical, endoscopic, radiological, and histological criteria according to the literature^11–13^. There were no age restrictions; the study included all age groups. The exclusion criteria were lack of main variables, such as any type of IBD (CD, UC, or unclassified IBD (IBD-U) and duplicate data.

### Clinical and sociodemographic variables

The variables from the registration data included date of birth; current age; type of healthcare service utilized; ethnicity (as personally declared); sex; education; medical history of comorbidities; smoking status (never smoked, current smoker, or past smoker); city/state of outpatient care; type of IBD (CD, UC, or IBD-U); age at symptom onset, diagnosis, and registration; Montreal classification^14^; disease severity; initial manifestations; extraintestinal manifestations (EIMs); presence of fistulas; comorbidities; and body mass index (BMI). Surgeries related to IBD, such as intestinal resections, placement of setons, colectomy due to intractable disease activity or neoplasia, and surgical complications (including complications related to short bowel syndrome or fecal incontinence) were also included. Information about previous hospitalizations (related to disease activity or IBD complications such as infectious diseases) and previous or current use of medications were recorded. In addition, data on family history of IBD or other immune-mediated diseases/cancers, including the degree of kinship, were also collected.

### Outcome variables

Disease severity was characterized by previous hospitalization, use of biologics, and need for surgery in patients with CD and UC. Complications such as disease activity, pancreatitis, or infectious diseases (herpes simplex or zoster, tuberculosis, upper airway infection, fungal infection, pneumonia, and urinary infection) were also considered for patients with CD. For CD, the independent variables for the final model were the Montreal Classification (L-disease location, B-disease behavior), age at diagnosis (1–20 y, 21–40 y, 41–60 y, and 61–88 y), sex (male vs. female), smoking status (current vs. past), comorbidities (yes vs. no), and rheumatic EIMs (yes vs. no). For UC, the independent variables were the extent of the disease, age at diagnosis (1–20 y, 21–40 y, 41–60 y, and 61–88 y), sex (male vs. female), smoking status (current vs. past), comorbidities (yes vs. no), rheumatic EIMs (yes vs. no), and presence of a familial history of colorectal colon cancer (yes vs. no).

### Statistical analysis

Data are expressed as mean ± standard deviation or median (range) for continuous variables and as frequency (proportion) for qualitative variables. Statistical analysis was performed using the Poisson regression model to obtain the raw and adjusted frequency ratios^15^. For patients with UC, four models were constructed based on four independent dependent variables: history of colectomy, colorectal cancer, hospitalization, and use of biologics or small molecules. The initial regression model was theoretically conceived as having as independent variables such as extensive colitis, proctitis, age at diagnosis, EIMs, comorbidities, BMI, smoking status, sex, and familial history of colon cancer. However, BMI was removed because of numerical insufficiency. Four models were constructed for patients with CD, based on four separately dependent variables: previous surgery, presence of complications (as described above), use of biologics, and hospitalization. As initial independent variables, the models considered the behavior of the disease (B2-stricturing; B3-penetrating), ileal location, colonic location, age at diagnosis, EIMs, comorbidities, smoking status, and sex. For all models, in assessing the contribution of each independent variable to the respective regression model, the module of the percentage difference was calculated for each exponentiated Beta of the regression (frequency ratio) compared with the value 1 as a reference. Patients with IBD-U were excluded because of the small sample size. A value of 5% was set as the minimum permanence value of the variable in the model. The goodness of fit of the models was assessed using the Akaike Information Criterion^16^ and residual analysis. To assess the assumption of non-overdispersion, the ratio of the deviance residuals to their degrees of freedom^17^ was used. Because the sampling plan was non-probabilistic, *P*-values or confidence intervals were not calculated owing to the lack of stability in the standard error estimates^18,19^. The global variance inflation factor (GVIF) was used to evaluate the presence of collinearity, assuming a GVIF < 5% with its absence^20^. Finally, the profile-predictive models were calculated from the final Poisson model equation for models with at least one remaining independent variable. Analysis was performed using the statistical package R (version 4.2.2)^21^ in Linux Mint version 21. The statistical review of the study was performed by biomedical statistician.

### Ethical considerations

All procedures performed in studies involving human participants were in accordance with the ethical standards of the institutional and/or national research committee and with the 1964 Helsinki Declaration and its later amendments or comparable ethical standards. This study was approved by the Local Research Ethics Committee, (CAAE: 71343417.2.1001.5629) and by all respective boards from the participating centers (listed in the Declarations). All participants received explanations about the study aims and expected results, having been enrolled in the study only after signing the informed consent term.

## Results

A total of 1179 patients were included: 600 (51%) with UC, 568 (48%) with CD, and 11 (0.9%) with IBD-U. Table 1 shows the clinical and demographic characteristics of the patients with IBD. A total of 108 patients were excluded due to inconsistence data.

**Table 1:** Clinical and demographic variables of IBD patients.

	CD	UC	U-IBD	IBD
No of patients (%)*	568 (48)	600 (51)	11 (0.9)	1179
Schooling				
Illiteracy	3 (0.8)	11 (2.9)	1 (1.25)	14 (1.8)
PE	41 (10.4)	111 (29.7)	6 (0.75)	153 (12.9)
LSE	163 (41.3)	134 (35.8)		300 (25.4)
USE	173 (43.3)	108 (28.9)	1 (1.25)	284 (24)
PSTE	15 (3.9)	10 (2.6)		26 (3.2)
Race				
Caucasian	367 (72)	426 (74.6)	8 (72)	815 (73)
Brown	117 (22.9)	119 (20.8)		50 (4.5)
Black	26 (5.1)	22 (3.9)	3 (28)	242 (22)
Others		4 (0.7)		
Females (%)	289 (51.1)	404 (67.4)	6 (54.5)	747 (59)
Mean age at diagnosis, years (range)	32 (14)	37 (15)	34 (14)	34 (14.7)
Mean time to diagnosis, years (range)	1.06 (2.82)	1.64 (3.25)	1.54 (2.16)	2.1 (0.3)
Smoking status (%)				
Never	455 (86.7)	493 (84.1)		963 (85.3)
Current	28 (5.3)	18 (3.1)		48 (4.3)
Former	42 (8)	75 (12.8)		118 (10.5)
BMI				
Malnourished	22 (6.7)	6 (1.6)		28 (3.9)
Normal weight	158 (48)	171 (46)	1 (16)	336 (47.2)
Overweight	94 (28.6)	122 (32.8)	5 (84)	220 (30.9)
Obese	55 (16.7)	73 (19.6)		128 (18)
Age at onset				
A1	47 (9.5)			
A2	311 (63.1)			
A3	135 (27.4)			
Disease location				
L1	182 (32)			
L2	151 (26.7)			
L3	234 (41.2)			
L4	19 (3.4)			
Disease behavior (%)				
B1	182 (32)			
B2	151 (26.7)			
B3	64 (11.3)			
p	88 (15.5)			
Disease extent				
UC (%)		134 (23.7)		
E1		170 (30)		
E2		262 (46.3)		
E3				

*CD* Crohn’s disease; *UC* ulcerative colitis; *U-IBD* unclassified inflammatory bowel disease; *B1* non-stricturing, non-penetrating; *B2* stricturing; *B3* penetrating; *PE* primary education; *LSE* lower secondary education; *USE* upper secondary education; *PSTE* post-secondary Tertiary education.

Regarding the initial symptoms, 42% presented with diarrhea, 38% with abdominal pain, and 20% with weight loss. The age at IBD symptom onset ranged from 1 to 87 years (32.3 ± 14.4) and the time from the beginning of the symptoms to diagnosis was 3.85 years. According to the Montreal Classification of CD, most patients were diagnosed between 17 and 40 years of age (63.1%), the main location was ileocolonic region (41.2%), and the main behavior was non-stricturing and non-penetrating (32%). Among patients with UC, 46.3% presented with extensive colitis, 30% had left-sided colitis, and 23.7% had proctitis.

According to BMI, 3.9% were malnourished, 30.9% were overweight, and 18% were obese. The main EIMs were rheumatic (21%), dermatological (4.2%), hepatic (2.6%), nephrological (2.1%), and ophthalmological (0.9%).

The Southeast region of Brazil, comprising the states of São Paulo, Rio de Janeiro, Minas Gerais, and Espírito Santo, had the highest percentage of patients included in the study (72.3%), followed by the Midwest (13.8%), Northeast (6.9%), South (4.9%), and North (1.9%).

Regarding medical treatment, 72.4% of patients were using or had previously received biologics (34.3% infliximab, 21.9% adalimumab, 7.2% vedolizumab, 6.5% ustekinumab, 1.7% certolizumab pegol, and 0.5% golimumab), 73.1% salicylates, 51.6% immunosuppressors, and 0.9% tofacitinib (Table 2).

**Table 2:** Previous and current medical treatment distribution among patients with inflammatory bowel disease (n = 1179).

Medical treatment	n (%)
Salicylic derivatives	862 (73.1)
Immunossupressants	609 (51.6)
Azathioprine	578 (49.0)
Methotretaxe	25 (2.1)
6-Mercaptopurine	6 (0.5)
Corticosteroids	306 (25.9)
Biological Therapy	854 (72.4)
Infliximab	405 (34.3)
Adalimumab	259 (21.9)
Certolizumab	21 (1.7)
Golimumab	7 (0.5)
Vedolizumab	85 (7.2)
Ustekinumab	77 (6.5)
Tofacitinib	11 (0.9)

Of those who underwent surgery (n = 312, 26.5%; elective vs. urgent/emergency: 54% vs. 46%), 240 (76.9%) had CD, 69 (22.1%) had UC, and 4 (1%) had U-IBD. Most procedures (80%) involved an open laparotomy, whereas 20% were laparoscopic. In total, 227 (72.7%) were abdominal (8.5% colectomy, 6.1% enterectomy, 1.2% diagnostic laparotomy, and 1.2% appendectomy, among others) and 76 (24%) were perianal (6% seton placement, 8% abscess drainage and curettage, and 4% fistulotomy, among others). Complications related to surgery were more prevalent in patients with CD (CD vs. UC: 45.3% vs. 19.3%). Almost 30% of the patients underwent more than one surgery.

At least one complication was reported in 62% of the patients; most of them were infectious disorders requiring prolonged hospitalization. Comorbidities were present in 72% of patients, and the most prevalent was high blood pressure 8.3%/3.3%, obesity 0.8%/0.5%, diabetes 0/0.3%, heart failure 0/0.3%, thrombosis 0.3%/0.6% and nephrolithiasis 2.3%/1.4%, in CD and UC patients, respectively.

### Clinical factors associated with severity in patients with CD

Table 3 shows the adjusted frequency ratio (FR) for disease severity in patients with CD. The presence of rheumatic EIMs was associated with the use of biological therapy (FR > 1.1). Patients’ presenting behavior, according to the Montreal classification, of B2 and B3; colonic location; and presence of rheumatic EIMs demonstrated a higher risk of disease complications (FR > 1.1). Frequency ratios indicated the absence of a contribution of the variables to the model for hospitalization and surgery.

**Table 3:** Clinical factors associated with biological therapy, hospitalization, abdominal surgery, and presence of complications in patients with CD (N = 448).

	Biological therapy (FR)	Hospitalization (FR)	Abdominal surgery (FR)	Complications (FR)
Montreal classification				
B2 behavior	1.06	1.00	1.03	**1.92**
B3 behavior	–	0.96	1.03	**1.26**
Location L1	–	1.05	1.04	0.82
Location L2	–	1.07	1.04	**1.10**
Age at diagnosis				
1–20 years	1.00	1.00	1.00	1.00
21–40 years	0.93	0.99	1.02	0.80
41–60 years	0.79	1.02	1.01	0.83
61–88 years	0.80	1.04	1.03	0.60
Rheumatic EIM (yes vs*.* no)	**1.11**	0.96	0.99	**1.34**
Comorbidity (yes vs*.* no)	–	1.00	1.01	0.90
Smoking (current vs. past)	0.91	0.95	0.98	0.79
Sex (male vs. female)	1.07	0.97	0.99	1.09

*FR* adjusted frequency ratio; *EIM* extraintestinal manifestation; *CD* Crohn’s disease.

E.g. Significant values are in [bold].

From the final adjusted model, it was possible to obtain the association between the different profiles of patients with CD and the need for biologics. Thus, the most associated patient profile had stricturing disease (B2 behavior), young age at diagnosis, presence of rheumatic EIMs, male sex, and no smoking/no history of smoking status, with an expected average value of 50.24% of the need for biological therapy. The profile most associated with the presence of complications was stricturing or penetrating behavior, not having an ileal location, having a colonic location, being young, having a rheumatic EIM, not having a comorbidity, not smoking, and being male. For such a profile, there is an expected 56% chance of disease complications.

### Clinical factors associated with severity in patients with UC

Table 4 shows the adjusted FR for disease severity with respect to UC. The presence of extensive colitis, rheumatic EIMs, male sex, and a familial history of colorectal cancer were associated with the necessity of biological therapy (FR > 1.1). Hospitalization was not associated with any of the variables studied. Regarding the need for colectomy, the associated variables were the presence of extensive colitis, rheumatic EIMs, comorbidities, and male sex (FR > 1.1).

**Table 4:** Clinical characteristics associated with biological therapy, hospitalization, and colectomy in patients with UC (N = 519).

	Biological therapy (FR)	Hospitalization (FR)	Colectomy (FR)
Disease location			
Proctitis	0.57	1.06	–
Extensive colitis	**1.77**	1.03	**11.1**
Age at diagnosis			
1–20 years	1.00	1.00	1.00
21–40 years	0.77	1.00	0.93
41–60 years	0.61	1.02	0.82
61–88 years	0.66	0.98	0.45
Rheumatic EIM (yes vs. no)	**1.49**	1.00	**1.41**
Comorbidity (yes vs. no)	0.86	1.01	**1.20**
Smoking (current vs past)	0.88	1.03	0.96
Sex (male vs. female)	**1.15**	0.99	**1.31**
Familial history of CRC (yes vs. no)	**1.50**	0.93	–

*FR* adjusted frequency ratio; *EIM* extraintestinal manifestation; *UC* ulcerative colitis; *CRC* colorectal cancer.

E.g. Significant values are in [bold].

From the final adjusted model, we suggest that patients with extensive colitis, young age at diagnosis, history of colorectal cancer in the family, presence of rheumatic EIMs, male sex, absence of comorbidities, and nonsmoking status had an expected mean value of 55.64% for the need for biological therapy. We also suggest that patients with extensive colitis, young age at diagnosis, presence of rheumatic EIMs, male sex, presence of comorbidity, and non-smoking status presented an expected mean value of 20.4% for the need for colectomy.

## Discussion

This study is the first cohort of patients with IBD from the Brazilian National Registry of Patients conducted by the GEDIIB in Brazil. Epidemiological studies are important knowledge tools for a given population aiming for future interventions. Studies with continuous inclusion of patients in their database can generate interesting results in the short, medium, and long terms and can help society, medical organizations, and the government in planning public policies to serve this specific population.

A recent increase in the incidence of IBD has been described in the literature, mostly in Asia^22^ and Latin America^23^, although a higher prevalence has also been reported in Africa^24^. In absolute numbers, the burden of IBD in developing countries may be greater than that of the combined burden in Europe and North America^25^, which increases costs for the healthcare system, as in most developing countries. In addition, early diagnosis is still lacking, with patients receiving a diagnosis at a later stage with complications. The number of preventable surgeries, outdated treatments (as 5-ASA for CD and corticosteroid use in maintenance therapy), and complications (such as neoplasia) compromise a patient's quality of life.

In Latin America, there are few epidemiological reports from national databases. Juliao-Banos et al.^26^ examined the epidemiological trends of IBD in Colombia using a national database of 33 million adults, encompassing 97.6% of the Colombian population. This study calculated the incidence and prevalence of UC and CD from 2010 to 2017 and examined the epidemiological trends according to urbanicity, demographics, and region. In addition, the IBD phenotype (Montreal Classification), prevalence of IBD-related surgeries, and types of IBD medications prescribed to adult patients attending a regional IBD clinic were assessed. Using a nationally representative sample and a regional clinic cohort, they found that UC is more common in Colombia and is increasing in urban regions. There were 649 patients with IBD in the clinical cohort: 73.7% with UC and 24.5% with CD. The mean ages at diagnosis of CD and UC were 41.0 years and 39.9 years, respectively. Most patients with UC developed extensive colitis (43%), whereas most patients with CD developed ileocolonic disease. A total of 16.7% of patients with CD had perianal disease. Patients with CD received more biologics than those with UC.

The National Registry from GEDIIB aims to quantify the real prevalence of IBD in the Brazilian territory as well as to identify the differences between the geographic regions^27^ of the country, given the territorial dimension of the same. Our sample demonstrated a predominance of extensive colitis in UC but had a higher percentage of patients with CD (48%). The mean age at the time of IBD diagnosis was 32 years in Brazil and 40 years in Colombia. The time from the beginning of the symptoms to diagnosis was approximately 3.8 years, almost the same as that found by Nobrega et al.^28^ and better than that reported by Fróes et al.^27^, who found a delay of 5 years between the reports of the first symptoms and the diagnosis of CD in Rio de Janeiro.

Long-term population studies may have a lower number of patients in their first publications, and it would be interesting to compare population profiles over time. The ECCO-EpiCom 2011 inception cohort analyzed the differences in disease phenotype, medical therapy, surgery, and hospitalization rates during the first year after diagnosis. A total of 258 patients with CD, 380 with UC, and 71 with unclassified IBD were included. Overall, 178 (25%), 460 (65%), and 71 (10%) patients were diagnosed in Eastern Europe, Western Europe, and Australia, respectively. During the first year after diagnosis, surgery and hospitalization rates were significantly higher in patients with CD in Eastern Europe than in those in Western Europe and Australia. In contrast, significantly more patients with CD were treated with biologics in Western European and Australian centers^29^. Our sample included 1179 cases, although modest, a larger number than in the ECCO-EpiCom 2011 inception and Colombia papers, with patients mostly found in the southeastern region of Brazil.

Our study included a representative sample of five regions of Brazil with retrospective and prospective data. It is important to note that the findings presented here reflect only initial data regarding the country's higher number of patients with IBD, as shown by Quaresma et al.^3^. According to their data, IBD has recently reached a prevalence of more than 60 cases per 100,000 inhabitants in Brazil, depending on the degree of urbanization in the region. Therefore, IBD is no longer considered rare. The results of regional studies also show a south-north gradient, with a higher incidence in urbanized areas in the South/Southeast region of the country^2,3,5,30^. In the states of São Paulo and Espírito Santo^30,31^, prevalence rates of IBD were 52.6/100,000 and 38.2/100,000, respectively in contrast with 12.8/100,000 in the state of Piaui^32^, localized in the northern part of the country, with lower human development index.

To date, most studies in Brazil have analyzed public health data from University Hospitals or the Unified Health System (DATASUS), which is an open-access population-based health and disease registry that contains information from the national unified health system (SUS) through records of hospital admissions, outpatient procedures, consultations, and IBD-related medication dispensing, searched according to the International Statistical Classification of Diseases and Related Health Problems, Tenth Revision (ICD-10), with codes K50 for CD and K51 for UC^2,5,30,31,33^. However, these studies lacked information on private services, which may have led to underestimation. As supplementary health is responsible for approximately 25% of the country's health coverage^34^, a National Registry can contribute to a more accurate epidemiological profile of IBD in the country. In this first analysis of the National Registry results, almost the same number of patients were followed up at private and public (academic or not) centers, suggesting that a relatively high proportion of patients can afford private health insurance. A National IBD Database with inputs from most gastroenterologists and proctologists of reference for IBD will greatly contribute to the establishment of a powerful, accurate, and dynamic tool for the study of IBD.

Our data are in line with those reported in the literature, considering the risk factors related to severe disease. After performing adjustments, we found that severe disease, defined as the need for hospitalization, surgery, and the use of biologics for UC, was associated with the presence of extensive colitis, rheumatic EIMs, male sex, comorbidities, and a familial history of colorectal colon cancer. Regarding CD, severe disease was associated with Montreal phenotypes B2 and B3, colonic location, and the presence of rheumatic EIMs. Younger age at initial diagnosis, the presence of perianal lesions, ileal involvement, smoking, and the need for corticosteroid therapy are the major predictors of a disabling disease or change of behavior to a more aggressive disease as reported by Blonski et al.^35^. Romberg-Camps et al.^36^ showed that in CD, small bowel location, stricturing disease, and young age predicted disease recurrence, whereas in UC, extensive colitis and older age at diagnosis were negative prognostic predictors. Recently, Sacramento et al.^37^ reported the variables associated with moderate-to-severe CD progression. These results are also in line with our findings, demonstrating perianal disease, stricturing or penetrating behavior, and ileocolonic localization. Age of < 40 years at diagnosis (81.3% vs. 62.0%, *P* = 0.004), upper gastrointestinal tract involvement (21.8% vs. 10.3%, *P* = 0.040), and perianal disease (35.9% vs. 16.3%, *P* < 0.001) were also associated with the use of immunobiological agents.

Almost half the patients in this study were overweight or obese. This is in line with studies linking environmental factors, especially diet, to an increased risk of immune-mediated diseases^38–40^. In Brazil, the increased consumption of ultra-processed foods over the last few decades has contributed to the burden of obesity^41,42^. Between 1974 and 2009, the prevalence of obesity in children aged 5–9 years increased from 2.9 to 16.5% among boys and from 1.8 to 11.8% among girls. For adolescents (aged 10–19 years), the prevalence varied from 0.4 to 5.9% in males and from 0.7 to 4.0% in females during the same period. In adults aged 20 years or older, the obesity prevalence increased by more than eightfold (2.8–22.8%) among men and threefold (8.0–30.2%) among women from 1974 to 2019^41,42^. The gradual weakening of traditional food patterns, based on fresh or minimally processed foods, with the concomitant increase in the consumption of ultra-processed foods, contributes to immune dysregulation of the intestinal microbiome in those with a genetic propensity for IBD and development of the disease. This is an important point of discussion as many healthcare professionals do not recognize IBD in non-malnourished patients. However, it is well known that being overweight or obese is associated with sarcopenia and vitamin deficiencies, possibly contributing to the worst prognosis in IBD.

Until 2020, Brazil had unequal access to medication for CD and UC, as demonstrated by Vilela et al.^43^, with a higher number of physicians reporting difficulty in accessing or releasing medicines for UC than for CD. Martins et al.^44^ also observed that the complication rate among patients with UC undergoing therapy available in the National Health System from 2011 to 2020 was higher among those receiving only conventional therapy. No biological therapy was available for UC in the public system until 2020, and only anti-TNF therapy was available for moderate-to-severe CD. Among patients from private services, only those with moderate-to-severe CD had access to anti-TNFs, anti-integrins, and anti-interleukins. The publication of national epidemiological studies^2,3,5,30–33,37,44–48^, physicians´ perceptions of IBD in Brazil^43^, and studies on the socioeconomic impact of IBD, including absence from work^27,49,50^, served to alert the authorities and resulted in the incorporation of biological therapy for UC in the public sphere in 2020 and in the private system in 2021.

The National Patient Registry, organized by the GEDIIB, is a prospective study in its beginning. An important limitation of this study is that once the registry was initiated during the COVID-19 pandemic, it did not include a large number of patients. However, as it includes data from the entire national territory, and it comprises a data registry with continuous inclusion of patients in their database, which can generate interesting results in the short, medium, and long terms and help society, medical organizations, and the government in planning public policies to serve this specific population with IBD, a major gastrointestinal disease.

## Conclusion

To date, no epidemiological study with public and private data has covered the entire Brazilian territory considering the clinical aspects associated with IBD severity. The results obtained from the ongoing registry will be fundamental for improving the information quality in a country with continental dimensions, such as Brazil. The greater the number of qualified participating researchers from different regions of the country, the greater the representativeness of the data, which may greatly help direct government actions on behalf of patients with IBD.

## Data Availability

Data, analytical methods, and study materials are available to other researchers upon specific request. Please contact the corresponding author.
